# Effects of Light Quality on Morphology, Enzyme Activities, and Bioactive Compound Contents in *Anoectochilus roxburghii*

**DOI:** 10.3389/fpls.2017.00857

**Published:** 2017-05-23

**Authors:** Shenyi Ye, Qingsong Shao, Mengjie Xu, Shuailing Li, Mei Wu, Xin Tan, Liyang Su

**Affiliations:** ^1^State Key Laboratory of Subtropical Silviculture, Zhejiang A & F UniversityHangzhou, China; ^2^Department of Traditional Chinese Medicine, Zhejiang A & F UniversityHangzhou, China; ^3^Key Laboratory of Plant Secondary Metabolism and Regulation of Zhejiang ProvinceHangzhou, China; ^4^Flower Research Institute, Jinhua Academy of Agricultural SciencesJinhua, China

**Keywords:** *Anoectochilus roxburghii*, light quality, morphology, enzyme activities, bioactive compounds

## Abstract

The aim of this study was to investigate the effects of light quality on the morphological traits, leaf anatomical characteristics, antioxidant enzyme (superoxide dismutase, catalase, and peroxidase) activities, photosynthetic pigments content, and bioactive compounds (phenols, flavonoids, and polysaccharides) content in *Anoectochilus roxburghii*. Plants of *A. roxburghii* were grown under light filtered through four differently colored films for 8 months. The four treatments were red film (RF), blue film (BF), yellow film (YF), and colorless plastic film (control, CK). Compared with the *A. roxburghii* plants in CK, those in the BF treatment showed significantly greater stem diameter, fresh weight, leaf area, stomatal frequency, chlorophyll content (Chl a, Chl b, Chl a+b), antioxidant enzyme activities, and active compound (polysaccharides, flavones) content. The plants in the RF treatment showed the greatest plant height and phenolics contents. These results show that growing *A. roxburghii* plants under blue film is a useful technique to improve quality. This technique is conducive to achieving large-scale sustainable production of high-quality plant materials.

## Introduction

*Anoectochilus roxburghii* (Orchidaceae) is a valued plant species in many Asian countries, where it is used for ornamental, culinary, and medicinal purposes. It is a widely used and popular functional food with several beneficial effects, such as its notable curative effects of clearing heat and cooling the blood, eliminating dampness, and detoxification. Various health products and foods can be produced from *A. roxburghii*, for example, health beverages, snacks, and soups. Because *A. roxburghii* is rich in polysaccharides, amino acids, alkaloids, flavonoids, and organic acids, it has been used to prevent and treat diabetes, hyperliposis, hepatitis, and tumors. Nevertheless, wild *A. roxburghii* resources are dwindling as a result of its specific growth conditions, slow growth rate, low seed germination rate, and long-term excavation (Lv et al., 2015). Thus, to meet the growing demands for this plant from herbal and functional food industries, and to avoid overexploitation of the wild resource, it is now cultivated instead of being harvested from wild populations.

Light is the most important factor affecting plant growth, with changes in irradiance affecting plant growth, morphology, various aspects of physiology, and plant productivity. In *A. roxburghii*, chloroplast ultrastructure in leaves developed better under 30% irradiance. It suggested that *A. roxburghii* increased levels of chloroplasts, grana, and grana lamellae, and higher POD and SOD activities to adapt shade conditions (Shao et al., 2014). Nevertheless, the effects of light quality are more complex. Plant species differ in their responses to light quality, but red and blue light generally have the strongest effects on the plant growth. The plant height of *Tagetes erecta* L. and *Salvia miltiorrhiza* Bunge was greater under blue light treatment than under red and fluorescent white-light treatments (Heo et al., 2002). Whereas, red light had the strongest stimulatory effect on the weight and height of *Rehmannia glutinosa* (Gaertn.) DC. (Manivannan et al., 2015). However, the effects of light quality on *A. roxburghii* have not been systematically studied and analyzed. Thus, the hypothesis of this study was that *A. roxburghii* plants would grow better under monochrome film compared to colorless plastic film (CLF). The objectives of this study were to determine the effects of light quality on the physiology, photosynthetic pigments content, enzyme activities, and bioactive compounds content in *A. roxburghii*, and to identify which light color was optimum for plant growth. The final goal of the research was to give an optimal suggestion of light quality to make growers obtain maximum economic benefits through regulating greenhouse light environment, as well as achieve large-scale continuous production.

## Materials and Methods

### Plant Materials and Growth Conditions

**FIGURE 1:**
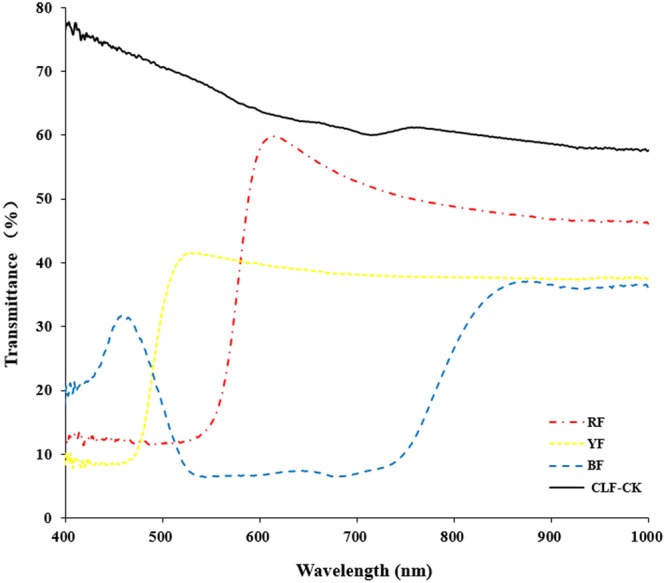
Transmittance of the filters (RF: red film, YF: yellow film, BF: blue film, CLF-CK: colorless film).

*Anoectochilus roxburghii* plants were collected in November 2015, and were maintained in a greenhouse at the Baicaoyuan test site of Zhejiang Agriculture and Forestry University, China (30°15′N, 119°43′E). The relative humidity in the greenhouse was 75%, and the temperature is in the range of 22–28°C. Plants were subjected to four different light quality treatments for 8 months. The treatments consisted of light filtered through red film (RF), blue film (BF), yellow film (YF), or CLF (Guofeilong trade co., LTD, Shenzhen, China) as the control (CK). Transmittance of the filters showed in **Figure 1**. Each treatment consisted of 10 pots with three replications. All plants were kept well-irrigated and were protected from bacterial pathogens and weeds.

### Morphological Observations

For measurements of morphological features, 15 plants were randomly selected from each treatment and the fresh weight, plant height, stem diameter, and leaf area were measured. Values shown in **Table 1** are the mean of 10 replicates.

**Table 1:** Effects of different color films on morphological traits of *Anoectochilus roxburghii.*

Treatment	Plant height (cm)	Stem diameter (mm)	Total fresh weight (g)	Leaf area (cm^2^)
RF	13.32 ± 0.05a	3.18 ± 0.01d	2.41 ± 0.06c	5.69 ± 0.10c
YF	13.04 ± 0.12a	3.24 ± 0.03c	2.45 ± 0.05c	5.66 ± 0.24c
BF	10.46 ± 0.10c	3.89 ± 0.03a	2.84 ± 0.04a	6.29 ± 0.14a
CLF-CK	12.06 ± 0.12b	3.46 ± 0.03b	2.63 ± 0.10b	6.06 ± 0.16b

*Values are the means ± SE of triplicate assays. Data in columns with the different letters are significantly different *P* < 0.05.*

### Leaf Anatomical Characteristics

*Anoectochilus roxburghii* leaf stomata were examined under a scanning electron microscope (SEM). Small leaf samples (ca. 2 mm × 5 mm) were collected and immediately immersed in cold 3% (v/v) glutaraldehyde in 0.05 M sodium cacodylate buffer (pH 7.2), and then fixed in 1% (v/v) osmium acid. The fixed samples were examined under a KYKY-EM3200 SEM (KYKY Technology Development Ltd., Beijing, China) (Yu et al., 2016).

### Photosynthetic Pigment Contents

Mature leaves were collected for determination of chlorophyll content (Chl a, Chl b, Chl a+b, Chl a/b). Chlorophylls were extracted by grinding leaves in 80% acetone in the dark at room temperature. The concentrations of chlorophylls were calculated based on the equations described by Porra and are expressed as mg g^-1^ FW (Porra, 2002).

### Enzyme Activities

Leaf samples (approximately 0.5 g) were collected from each treatment. The activities of peroxidase (POD), superoxide dismutase (SOD), and catalase (CAT) were determined as described elsewhere (Mahdavikia et al., 2017).

### Bioactive Compounds Content

Powdered samples (1 g) were accurately weighed and then extracted for 8 h in a Soxhlet extractor with ethanol–water solvent (85%, v/v) until the samples became colorless. The extract solution was concentrated and centrifuged.

#### Total Phenolics

A modified version of the Folin–Ciocalteu method (Costa et al., 2016) was used to quantify total phenolics, with gallic acid (Aladdin, Shanghai, China) as the standard. The absorbance of the solution at 760 nm was measured using a spectrophotometer. The yield of phenols was calculated. All samples were analyzed in triplicate.

#### Total Flavonoids

The flavonoids content was determined by the NaNO_2_-Al(NO_3_)_3_-NaOH method (Zhu et al., 2009). Rutin (Aladdin) was used as the standard. The absorbance of the solution was determined by visible spectrophotometry at 510 nm. The yield of flavonoids was calculated. All samples were analyzed in triplicate.

#### Total Polysaccharides

Total polysaccharides were extracted from *A. roxburghii* plants using the ethanol subsiding method (Liu et al., 2014). The polysaccharide concentration was determined using the phenol-sulfuric acid method. The absorbance of the solution was measured at 488 nm, and d-glucose (Aladdin) was used as the standard. The yield of polysaccharide was calculated. Samples were analyzed in triplicate.

### Statistical Analysis

Mean values of treatments were compared by one-way ANOVA using SPSS 22.0 software (SPSS, Chicago, IL, United States). The least significant difference test (LSD) was used to detect differences between means (*P* < 0.05). In figures and tables, values shown are mean ± standard error (SE).

## Results

### Morphological Traits

The different light qualities affected the morphological traits of *A. roxburghii.* Stem diameter, fresh weight, and leaf area were significantly higher in the BF treatment than in CK (**Table 1**). The largest stem diameter was in the BF treatment (3.89 mm), and the mean stem diameter differed significantly among BF, YF, RF, and CK. The highest fresh weight (2.84 g) was in the BF treatment, and the lowest fresh weights were in the RF and YF treatments. The leaf area was greater in the BF treatment (6.29 cm^2^) than in other treatments, but there was no significant difference in leaf area between the RF and YF treatments. These results indicate that BF enhanced the photosynthesis rate, reduced plant height, and increased the fresh weight of *A. roxburghii.*

### Leaf Anatomical Characteristics

**FIGURE 2:**
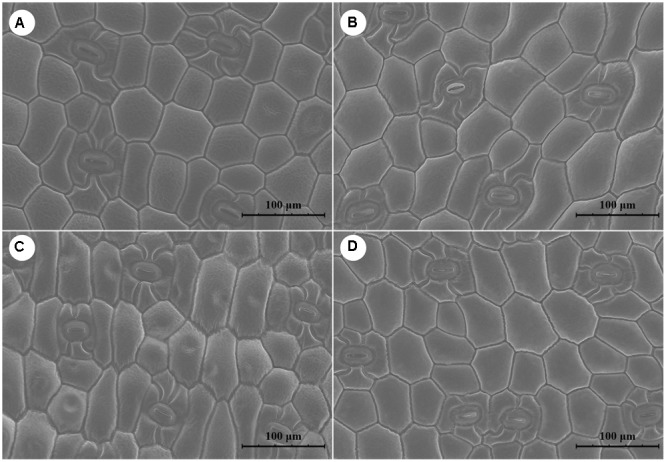
Scanning electron microscope (SEM) figure of leave surfaces of *A. roxburghii* plants. **(A)** *A. roxburghii* in CLF-CK treatment; **(B)** *A. roxburghii* in RF treatment; **(C)** *A. roxburghii* in YF treatment; **(D)** *A. roxburghii* in BF treatment.

Cells were longer and narrower in plants grown under RF and YF than in those in CK. However, the cell shape was the same in BF and CK (**Figure 2**). Overall, stomatal frequency was significantly higher in the BF and YF treatments than in the RF treatment and CK, but it did not differ significantly between the BF and YF treatments (**Table 2**). Stomatal length was smaller in the RF, BF, and YF treatments than in CK. However, stomatal width did not differ significantly among the treatments. The stomatal area (stomatal length × width; in μm^2^) under the different color film treatments was as follows: 969.27 (CK), 868.56 (RF), 816.43 (BF), and 763.56 (YF).

**Table 2:** Effects of different color films on stoma density and size of *A. roxburghii* leaves.

Treatment	Stomatal frequency (number/μm^2^)	Stomatal length (μm)	Stomatal width (μm)	Length × width (μm^2^)
RF	13.95 ± 0.04b	39.01 ± 0.57b	22.95 ± 0.33a	868.56 @ 44.58b
YF	16.74 ± 0.05a	33.22 ± 0.95d	23.61 ± 0.87a	763.56 @ 52.87c
BF	16.75 ± 0.03a	34.65 ± 1.00c	23.17 ± 0.50a	816.43 @ 27.28bc
CLF-CK	12.51 ± 0.08c	41.09 ± 0.19a	24.26 ± 1.50a	969.27 @ 67.20a

*Values are the means ± SE of triplicate assays. Data in columns with the different letters are significantly different *P* < 0.05.*

### Photosynthetic Pigment Content

Different light qualities significantly affected the chlorophyll content (**Table 3**). The Chl a, Chl b, and Chl a+b contents were higher in the BF treatment than in the RF and YF treatments, as compared with CK. The chlorophyll concentrations in the BF treatment were as follows: 1.48 (Chl a), 0.75 (Chl b), 2.23 (Chl a+b) mg⋅g^-1^ FW. This result indicated that BF strongly affected the photosynthetic system of *A. roxburghii*. The highest Chl a/b value (2.26 mg⋅g^-1^ FW) was in the YF treatment, and the lowest was in the BF treatment.

**Table 3:** Effects of different color films on photosynthetic pigments content of *A. roxburghii* leaves.

Treatment	Chl a (mg⋅g^-1^FW)	Chl b (mg⋅g^-1^ FW)	Chl a+b (mg⋅g^-1^ FW)	Chl a/b (mg⋅g^-1^ FW)
RF	1.10 ± 0.04c	0.51 ± 0.02c	1.61 ± 0.03c	2.16 ± 0.07b
YF	1.06 ± 0.02c	0.47 ± 0.03c	1.53 ± 0.03d	2.26 ± 0.04a
BF	1.48 ± 0.03a	0.75 ± 0.02a	2.23 ± 0.02a	1.97 ± 0.05c
CLF-CK	1.26 ± 0.02b	0.62 ± 0.01b	1.88 ± 0.02b	2.03 ± 0.03c

*Values are the means ± SE of triplicate assays. Data in columns with the different letters are significantly different *P* < 0.05.*

### Protective Enzyme System Activity

**FIGURE 3:**
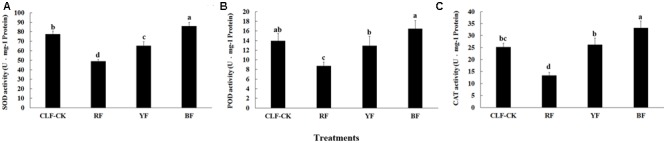
Effects of different color films on **(A)** SOD, **(B)** POD, and **(C)** CAT activity of *A. roxburghii.* Values are the means ± SE of triplicate assays. Data in columns with the different letters are significantly different *P* < 0.05.

After the 8-month treatment, the SOD, POD, and CAT activities were significantly lower in the RF treatment than in the other treatments. The activities of these enzymes were significantly higher in the BF treatment than in CK (**Figure 3**). The highest SOD activity (85.83 U⋅mg^-1^ protein) was in the BF treatment. The activity of POD differed significantly among the four treatments, and ranged from 8.73 (RF) to 16.45 U⋅mg^-1^ protein (BF). The highest CAT activity was 33.12 U⋅mg^-1^ protein (BF), and CAT activity did not differ significantly between the YF treatment and CK.

### Bioactive Compounds Content

The total flavonoid content was significantly higher in the BF treatment than in the other treatments, and ranged from 0.017 (YF) to 0.021 g⋅g^-1^ DW (BF) (**Table 4**). The total flavonoid content did not differ significantly between the RF and YF treatments. The total phenolics content was significantly higher in the RF treatment (0.018 g⋅g^-1^ DW) than in the other treatments, and differed significantly among the four treatments (**Table 2**). The total polysaccharides content in *A. roxburghii* plants differed among the treatments and ranged from 0.061 to 0.118 g⋅g^-1^ DW. The highest total polysaccharide content (0.118 g⋅g^-1^ DW) was in the BF treatment. These results showed that light quality strongly affected the accumulation of active compounds in *A. roxburghii*.

**Table 4:** Effects of different color films on active ingredients content of *A. roxburghii.*

Treatment	Polysaccharide (g⋅g^-1^ DW)	Flavone (g⋅g^-1^ DW)	Phenolic (g⋅g^-1^ DW)
RF	0.076 ± 0.0053c	0.017 @ 0.0001c	0.018 ± 0.0002a
YF	0.061 ± 0.0011d	0.017 @ 0.0003bc	0.009 ± 0.0003c
BF	0.118 ± 0.0019a	0.021 @ 0.0008a	0.010 ± 0.0001b
CLF-CK	0.097 ± 0.0039b	0.018 @ 0.0004b	0.005 ± 0.0001d

*Values are the means ± SE of triplicate assays. Data in columns with the different letters are significantly different *P* < 0.05.*

## Discussion

Light plays an important role in plant growth. In the last decade, many studies have focused on the effect of light quality on plant growth. Plants grow faster in low-light or dark conditions, and more slowly in bright light. This phenomenon reflects the ability of plants to respond to changes in the light environment to complete their life cycle and improve their biological yield. In rice, *OsHAL3* mediates light-controlled development, and its mechanism differs from that of other photoreceptors. The protein encoded by *OsHAL3* must form a trimer to function (Su et al., 2016). Light, especially blue light, leads to the disintegration of the trimer, resulting in protein inactivation, and light also inhibits the expression of its encoding gene. This dual inhibition by light slows cell division and eventually slows the growth of rice plants.

Plant species differ in their responses to light quality, but red and blue light generally have the strongest effects on the plant growth. Heo et al. (2002) reported that the plant height of *Tagetes erecta* L. and *Salvia miltiorrhiza* Bunge was greater under blue light treatment than under red and fluorescent white-light treatments. Su et al. (2014) reported that red light had an inhibitory effect on plant height, leaf area, and fresh weight of cucumber seedlings. Among several light treatments, blue light resulted in the largest leaf area of *Alternanthera brasiliana* (L.) Kuntze (Macedo et al., 2011). Red light had the strongest stimulatory effect on the weight and height of *Taraxacum officinale* (L.) Weber ex F.H.Wigg. (Ryu et al., 2012) and *Rehmannia glutinosa* (Gaertn.) DC. (Manivannan et al., 2015). Stevia plantlets had shorter stems and roots under blue light (Simlat et al., 2016). In our study, the morphological characteristics of *A. roxburghii* grown for 8 months were strongly influenced by light quality. Plants of *A. roxburghii* grown under BF had shorter, thicker stems, and the greatest total fresh weight and leaf area among all the treatments. The opposite trends were observed in the RF treatment.

Stomata regulate gas exchange and water loss in plants. Their opening and closing is influenced by many environmental factors, including light, CO_2_, and temperature. Among all these factors, light is the main environmental signal that controls stomatal movement. Usually, stomata are open in the light and closed in the dark. Blue light induces the guard cells to swell by activating their osmotic potential, leading to stomatal opening. The blue-light-induced stomatal opening response depends on the activation of a plasma membrane H^+^-ATP enzyme, the proton pump that produces the cell membrane potential. The blue light signal causes the voltage-dependent plasma membrane K^+^ channels to open, which enhances K^+^ and water flow into the guard cells, and finally forces the stomata to open (Schroeder et al., 2001; Shimazaki et al., 2007). Blue light resulted in reduced numbers of stomata in *A. brasiliana* (Macedo et al., 2011) and *Salvia splendens* Sellow ex Schult.. However, blue light led to more stomata in *T. erecta* (Heo et al., 2002) and all of the grape genotypes tested (Poudel et al., 2008). However, stomatal size in the grape genotypes did not differ significantly among the different light treatments. Simlat et al. (2016) reported similar results for *Stevia* plantlets. In our research, we observed that blue light led to the highest number of stomata in *A. roxburghii* among all of the light treatments. The stomatal length also differed significantly among the four light treatments, with the longest stomata in CK. However, stomatal width did not differ significantly among the four light quality treatments in this study.

Chlorophyll is one of the most important pigments in higher plants. It is the pigment responsible for capturing light for photosynthesis, which converts light energy into the chemical energy needed for plant growth. Therefore, chlorophyll is the key player in the interaction with light during the entire life cycle of plants. Light quality directly affects photosynthesis because of changes in chlorophyll content and composition. Xu et al. (2004) reported that in strawberry plants grown under different colors of plastic film, the treatments were ranked, based on highest chlorophyll content to lowest, as follows: RF > white film > YF > green film > BF. The chlorophyll a/b ratio was negatively correlated with the ratio of red to blue light. Galdiano et al. (2012) showed that red light strongly promoted chlorophyll b synthesis in *Cattleya loddigesii* Lindl.. Similar results were also reported for *R. glutinosa* and *Triticum aestivum* L. (Dong et al., 2014; Manivannan et al., 2015). However, Kobayashi et al. (2013) reported that the chlorophyll content in lettuce leaves was higher under blue light than under red light. Similar results were reported for *Toona sinensis* (Juss.) M.Roem. (Zhang et al., 2010). In this study, blue light had the strongest effect to stimulate chlorophyll accumulation in *A. roxburghii*. The markedly higher leaf chlorophyll content in the BF treatment than in the other treatments illustrated that *A. roxburghii* plants are able to maximize their light harvesting capacity under blue light.

In cells, CAT, SOD, and POD scavenge harmful free radicals. The O_2_^-^ produced in plants is removed by SOD and CAT, which protect plant cells against damage caused by free radicals and their derivatives. The activity of POD directly affects the metabolism and distribution of auxin (indole acetic acid, IAA), which controls plant growth and development. Strong POD activity enhances the oxidative decomposition of endogenous IAA, resulting in growth inhibition and a dwarf phenotype. Kim et al. (2013) reported that CAT and SOD activities in tomato leaves were higher under blue light than under a red light. Similar results were reported for *R. glutinosa* (Manivannan et al., 2015). In *Stevia* plantlets, CAT and POD activities were higher under blue light than under red light (Simlat et al., 2016), consistent with our results. These findings further confirmed that BF treatment benefits the growth and quality of *A. roxburghii* plants.

Light strongly affects the primary metabolism of plants, but it also affects the accumulation of secondary metabolites. Blue light promotes the accumulation of polysaccharides. This is achieved by increasing the Ca^2+^-CaM signal’s control of the photosynthetic apparatus or glucose metabolism (Lin, 2015). In *Dendrobium catenatum* Lindl., red light was shown to promote the accumulation of carbohydrates, thereby increasing the polysaccharide content (Lin and Lai, 2015). In contrast, blue light promoted polysaccharide accumulation in *Astragalus membranaceus* (Fisch.) Bunge, to a level 23.9% higher than that in the control (Ren et al., 2014). Our results were consistent with that finding, as blue light had the strongest stimulatory effect on polysaccharide accumulation in *A. roxburghii* among all of the treatments. In *Arabidopsis thaliana*, the blue light receptor cryptochromes (CRY1 and CRY2) and PhyA mediate responses to blue light to promote flavonoid biosynthesis and accumulation (Lin, 2009). In *A. membranaceus*, a blue light treatment resulted in the highest flavonoids content leaves, a level 51% higher than that in the control (Ren et al., 2014). In *A. roxburghii*, the flavonoids content was higher in the BF treatment than in the other treatments. Light promotes the accumulation of phenolic compounds, via increased production of malonyl CoA and coumaroyl CoA that serve as substrates for phenolics biosynthesis (Kim et al., 2006). Johkan et al. (2010) reported that blue light promoted the accumulation of phenolic compounds in *Lactuca sativa* L. seedlings. In sweet basil, the total phenolics content was lower in a blue light treatment than in a white light treatment (Shoji et al., 2011). In our study, the total phenolics content in *A. roxburghii* was higher under red light than under blue light.

## Conclusion

Filtering light through BF resulted in high-quality plants of *A. roxburghii* with the highest fresh weight, the most robust stem, the largest leaf area, and the highest stomatal frequency, as well as the highest photosynthetic pigment concentration and activities of antioxidant enzymes (CAT, SOD, and POD). The plants grown under BF also showed higher bioactive compounds contents, compared with the plants in other light treatments. Growing *A. roxburghii* plants under BF is a useful technique to improve quality. In terms of economic significance, this technique has the advantage of being low-cost for large-scale cultivation. Further studies are needed to explore the mechanism and interaction between bioactive compounds and light signal transduction pathways.

## Author Contributions

SY is responsible for the whole process of experimenting and writing the paper. QS provides experimental guidance. MW mainly assisted in the cultivation of experimental samples. MX, SL, XT, LS assisted in the main part of the experiment.

## Conflict of Interest Statement

The authors declare that the research was conducted in the absence of any commercial or financial relationships that could be construed as a potential conflict of interest.
